# Developmental Programming of Long Non-Coding RNAs during Postnatal Liver Maturation in Mice

**DOI:** 10.1371/journal.pone.0114917

**Published:** 2014-12-11

**Authors:** Lai Peng, Ariel Paulson, Hua Li, Stephanie Piekos, Xi He, Linheng Li, Xiao-bo Zhong

**Affiliations:** 1 Department of Pharmaceutical Sciences, School of Pharmacy, University of Connecticut, Storrs, Connecticut, United States of America; 2 Stowers Institute for Medical Research, Kansas City, Missouri, United States of America; Beckman Research Institute of City of Hope, United States of America

## Abstract

The liver is a vital organ with critical functions in metabolism, protein synthesis, and immune defense. Most of the liver functions are not mature at birth and many changes happen during postnatal liver development. However, it is unclear what changes occur in liver after birth, at what developmental stages they occur, and how the developmental processes are regulated. Long non-coding RNAs (lncRNAs) are involved in organ development and cell differentiation. Here, we analyzed the transcriptome of lncRNAs in mouse liver from perinatal (day −2) to adult (day 60) by RNA-Sequencing, with an attempt to understand the role of lncRNAs in liver maturation. We found around 15,000 genes expressed, including about 2,000 lncRNAs. Most lncRNAs were expressed at a lower level than coding RNAs. Both coding RNAs and lncRNAs displayed three major ontogenic patterns: enriched at neonatal, adolescent, or adult stages. Neighboring coding and non-coding RNAs showed the trend to exhibit highly correlated ontogenic expression patterns. Gene ontology (GO) analysis revealed that some lncRNAs enriched at neonatal ages have their neighbor protein coding genes also enriched at neonatal ages and associated with cell proliferation, immune activation related processes, tissue organization pathways, and hematopoiesis; other lncRNAs enriched at adolescent ages have their neighbor protein coding genes associated with different metabolic processes. These data reveal significant functional transition during postnatal liver development and imply the potential importance of lncRNAs in liver maturation.

## Introduction

As a vital organ of the digestive system, a matured adult liver plays a major role in nutrient homeostasis, including the synthesis, metabolism, and transport of carbohydrates, proteins, and fats. Bioactivation, detoxification, and filtration of compounds are other critical functions of the adult liver [1]. Venous blood from the stomach and intestine flows through the liver by the portal vein before entering systemic circulation. Thus liver is the first organ to encounter and deal with ingested drugs, environmental toxicants, and intestinal bacteria. However, these functions in adult liver are not mature yet in fetal and neonatal liver. Fetal liver is the major hematopoietic organ responsible for generating blood cells, and hematopoiesis is still active in liver even shortly after birth [2]. From neonatal to adult after birth, dramatic changes happen in liver to achieve the organ growth and functional transition from hematopoiesis to metabolism. Such functional transition has been implicated in clinical practice. Age-related sensitivity to drugs is at least partly attributable to differences in hepatic metabolic activity [3].

Functional transition over time during liver maturation is relied on finely programmed alteration of gene expression. Mouse has been served as a laboratory model to systematically study the alteration of gene expression in liver during development [4]. Mouse liver originates from the gut endoderm on embryonic day 8.5 when epigenetic markings, such as unwinding of the chromatin by FoxA transcription factors, contribute to the competence of embryonic liver development [5]. Signals, such as FGF-1, FGF-2, and BMP4 from the cardiac mesoderm, specify the foregut endoderm to begin expressing liver-specific genes. One day later, cells forming the hepatic endoderm assume a columnar morphology and are ready to form the liver bud. Expression of homeobox and prospero-related homeobox 1 genes is essential to the formation of liver bud. By embryonic day 15, hepatoblasts begin to differentiate into hepatocytes and bile-duct epithelial cells. HNF-6, HNF-1β, and the Notch/Jagged signaling pathways induce differentiation toward a biliary epithelial lineage, while HNF-4α followed by C/EBPα produces mature hepatocytes [6], [7]. The differentiation process is also driven by the secretion of oncostatin M by blood cells in fetal liver [8]. Although extensive researches have been done and advanced knowledge has accumulated on embryonic liver development, many aspects remain unknown in the postnatal liver development, such as the timeline of liver functional switch, the factors regulating liver growth, and the mechanisms driving the organ maturation.

Many previous studies have focused on examination of protein coding genes during liver development without a special attention on long non-coding RNAs (lncRNAs). lncRNAs are non-protein-coding RNA transcripts longer than 200 nucleotides [9]. They are alternatively spliced, contain 5′-capping and 3′-polyadenylation just like protein-coding RNAs, but have few or no open reading frames [10]. They either locate in the intergenic regions, called long intergenic noncoding RNAs (lincRNAs), or partially overlap with protein coding genes and can be transcribed from sense or antisense strand of the gene [11]. Comparative analysis has indicated that lncRNAs are evolutionarily conserved, especially at the promoter region [12]. The expression level of lncRNAs are usually lower than protein coding RNAs [11], [13], and they are expressed in tissue-specific and developmentally regulated manners [11], [13], [14]. Some specific lncRNAs are transcriptionally regulated by key transcription factors in a mechanism similar to their neighborhood protein coding genes [12]. lncRNAs have been demonstrated to serve as signal, decoy, guide or scaffold to regulate various biological processes [15], including the cell cycle [16], pluripotency [17], X-inactivation [18], cell differentiation [11], and organ development and maturation [19]–[21]. lncRNAs have also been associated to liver disease progress [22]–[24]. Yet whether lncRNAs are involved in regulation of postnatal liver development has not been explored before.

As first attempt in the current study, we used RNA-Sequencing (RNA-Seq) to quantify the liver transcriptome with a special focus on lncRNAs during the developmental period from perinatal stage to adult. RNA-Seq allows the analysis of the whole transcriptome with lower background noise, higher sensitivity, and a high degree of reproducibility compared to traditional technologies [25], [26]. More importantly, RNA-Seq quantifies the true abundance of RNA molecules in biological samples and enables the comparison of expression of all genes in multiple samples [27]. It also provides the chance for us to investigate the expression of lncRNAs during liver maturation. Our results revealed the dynamic transcriptional changes happened in liver during postnatal development with specific transition and maturation ages defined, and also suggested the potential importance of lncRNAs in liver maturation, which may facilitate future investigations to identify mechanistic roles of lncRNAs in regulation of gene expression during liver maturation.

## Materials and Methods

### Animals

Uses of animal were performed as previously described procedures [28]. Mice were housed according to the American Animal Association Laboratory animal care guidelines and were bred under standard conditions in the Laboratory Animal Resources Facility at the University of Kansas Medical Center. The use of these mice was approved by the Institutional Animal Care and Use Committee (IACUC) at the University of Kansas Medical Center.

### Total RNA extraction, sequencing library construction, and RNA-Seq

RNA extraction, library construction, and RNA-Seq were performed as previously described procedures [28].

### RNA-Seq data analysis

After the sequencing images were generated by the sequencing platform, the pixel-level raw data collection, image analysis, and base calling were performed by Illumina's Real Time Analysis software. The output bcl files were converted to qseq files by Illumina's BCL Converter 1.7 software and subsequently converted to FASTQ files for downstream analysis. The original bcl files were deposited into the Gene Expression Onmibus database with a series entry of GSE58827 (http://www.ncbi.nlm.nih.gov/geo/query/acc.cgi?acc=GSE58827). For the analysis of protein-coding and non-coding genes at all 12 ages, FASTQ files were aligned to mm9 genome with Tophat 1.4.1. Custom GTF was supplied to the program, which was compiled from Ensembl 66 genes and selected (non-overlapping) Noncode v3.0 models [29]. Cufflinks 1.3.0 [30] was used to quantitate expression levels with the same custom GTF file provided. The RNA abundance was expressed as the number of fragments per kilobase of exon per million reads mapped (FPKM). The output files in BAM format were analyzed by Cufflinks 2.1.1 to estimate the transcript abundance.

### Differential expression analysis

Statistically significant differential expression in this analysis were defined by ANOVA with the following criteria: 1) the gene mean FPKM >1 across the ages; 2) fold change for the average FPKM of the three replicates >1.5 between compared samples; 3) Benjamini-Hochberg adjusted p-values from t-test <0.05. The list of lncRNAs correlated with their neighboring protein-coding genes was selected using the following criteria: 1) The lncRNA was expressed with significant changes during development. The significant change of expression was detected using ANOVA with a threshold of adjusted p-value <0.05. 2) A neighboring protein-coding gene was expressed in the same developmental pattern as the lncRNA, and the genomic location between these two genes <10 kb. Go analysis was done for the neighboring protein-coding genes that were correlated with this list lncRNAs. Significantly enriched GO categories were selected with a false discovery rate (FDR) <0.05.

### Data Visualization and Statistics

For the visualization of developmental gene expression patterns in liver, protein-coding and non-coding genes were separated and hierarchically clustered (Pearson correlation distance, average linkage). ANOVA was used to test for significant difference in expression during development. P-values were adjusted using Benjamini-Hochberg algorithm with a threshold of 0.05. Protein-coding genes were from Ensembl 66, and non-coding genes included Ensembl 66 non-coding and Non-code non-overlapping annotations. Ensembl t/r/sn/sno/mi/misc-RNAs were removed from the final list of non-coding RNAs, and RNA-Seq used only poly-T beads selected RNAs for sequencing, so the list mainly contained lncRNAs. For Pearson correlation coefficient-based heat map visualization, the average FPKM of the three replicates at each age were used to calculate the Pearson's r values between different ages. The idiogram was made using the web application Idiographica [31].

## Results

### Transcriptome of protein-coding genes and lncRNAs in mouse postnatal liver development

**Figure 1:**
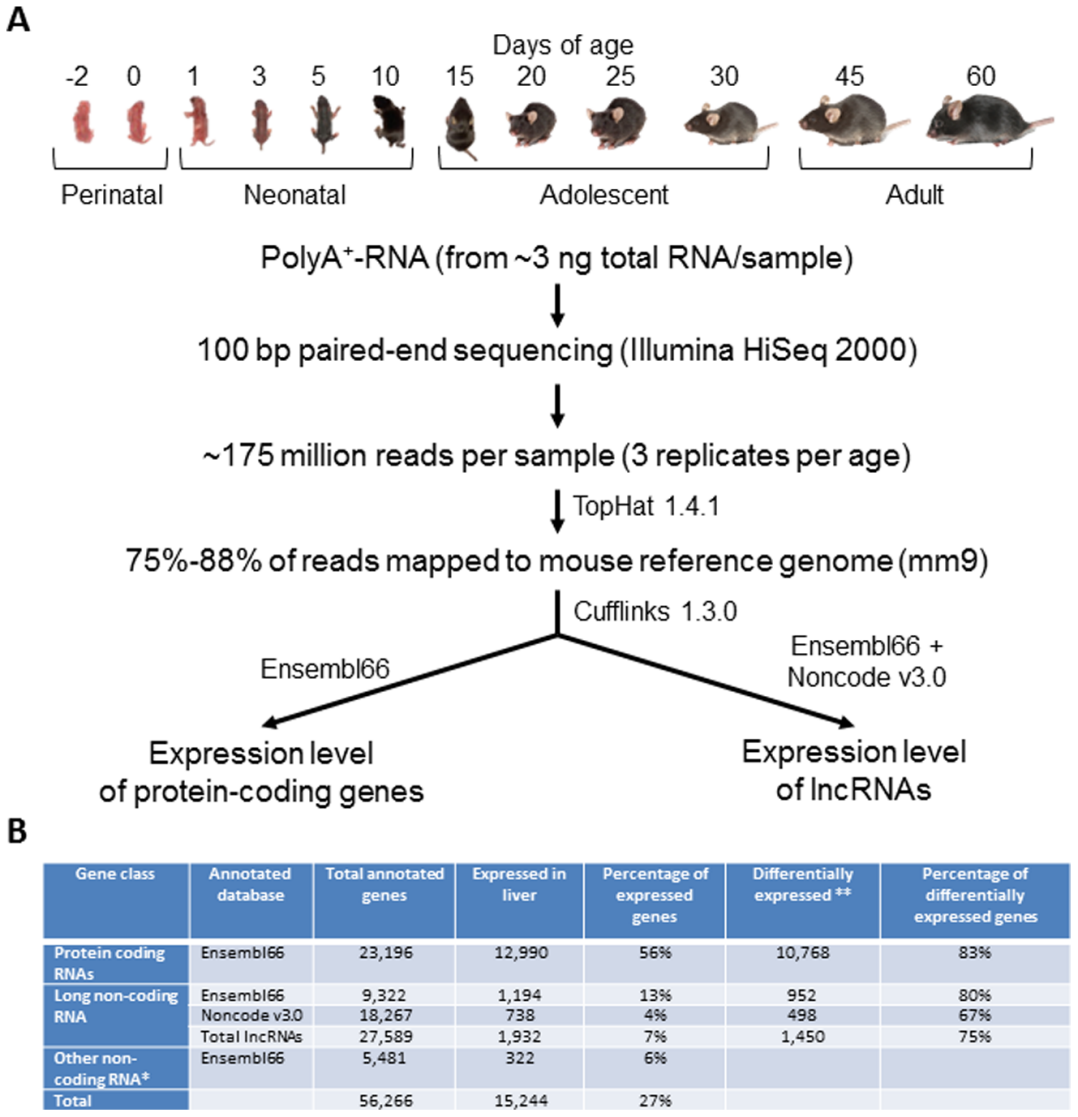
Identification of lncRNA in mouse liver. (A) Pipeline for gene annotation and identification of lncRNAs. (B) Number of Protein-coding genes and lncRNAs expressed during liver postnatal maturation. *Other non-coding RNAs include Ensembl66 annotated t-RNAs, r-RNAs, miRNAs, snRNAs, snoRNAs, and miscRNAs. **ANOVA was used to test for significant difference in expression during development. *P*-values were adjusted using Benjamini-Hochberg algorithm with a threshold of 0.05.

RNA-Seq generated an average of 175 million (from 172 to 179 million) 100 bp paired end reads per liver sample for the 36 samples from perinatal (Day -2 and 0), neonatal (Day 1, 3, 5, and 10), adolescence (Day 15, 20, 25, and 30), and adult (Day 45, and 60) (n = 3) with a mean of 83% (from 75% to 88%) of the reads mapped to the mouse reference genome (NCBI37/mm9). The mapped reads were annotated by Cufflinks 1.3.0 to protein-coding genes with Ensembl66 and non-coding genes with Ensembl66 and Noncode v3 as the reference annotation databases (**Fig. 1A**). Because the sequencing libraries were constructed from poly-T bead selected RNAs, the detected non-coding RNAs were mainly lncRNAs, but some small non-coding RNAs, such as tRNAs, rRNAs, snRNAs, snoRNAs, and miRNAs, were also detectable. FPKM (fragments per kilobase of exon per million reads mapped) is used to represent gene expression level for all annotated genes (**S1 Table**). The distribution of gene expression levels revealed that the majority of lncRNAs were expressed at a lower level than protein coding genes with a sharp distribution peak around FPKM = 1 in all 12 ages. As an example, distribution curves at day 5 are shown in **S1 Figure**.

When the genes with FPKM >1 in any age were considered as the genes expressed during liver postnatal maturation, as showed in Fig. 1B, a total of 15,244 (27%) among the 56,266 annotated mouse genes by both annotation databases were expressed in liver over the 12 ages, including 12,990 (56% of 23,196) protein-coding genes, and 1,932 (7% of 27,589) lncRNAs. Numbers of expressed genes for both protein-coding genes and lncRNAs decrease when liver is more mature (**Table 1**), implying that matured liver has more specific functions than immature liver.

**Table 1:** Number of expressed protein-coding genes and lncRNAs at each developmental age.

Developmental stage	Perinatal		Neonatal				Adolescent				Adult	
Day	−2	0	1	3	5	10	15	20	25	30	45	60
Protein-coding genes	11,355	11,283	11,805	11,635	11,507	11,393	10,850	10,297	10,056	9,806	9,466	9,786
lncRNAs	1,617	1,383	1,491	1,420	1,364	1,365	1,193	1,157	1,224	1,176	1,128	1,155

### Developmental changes of protein-coding genes and lncRNAs in mouse postnatal liver development

**Figure 2:**
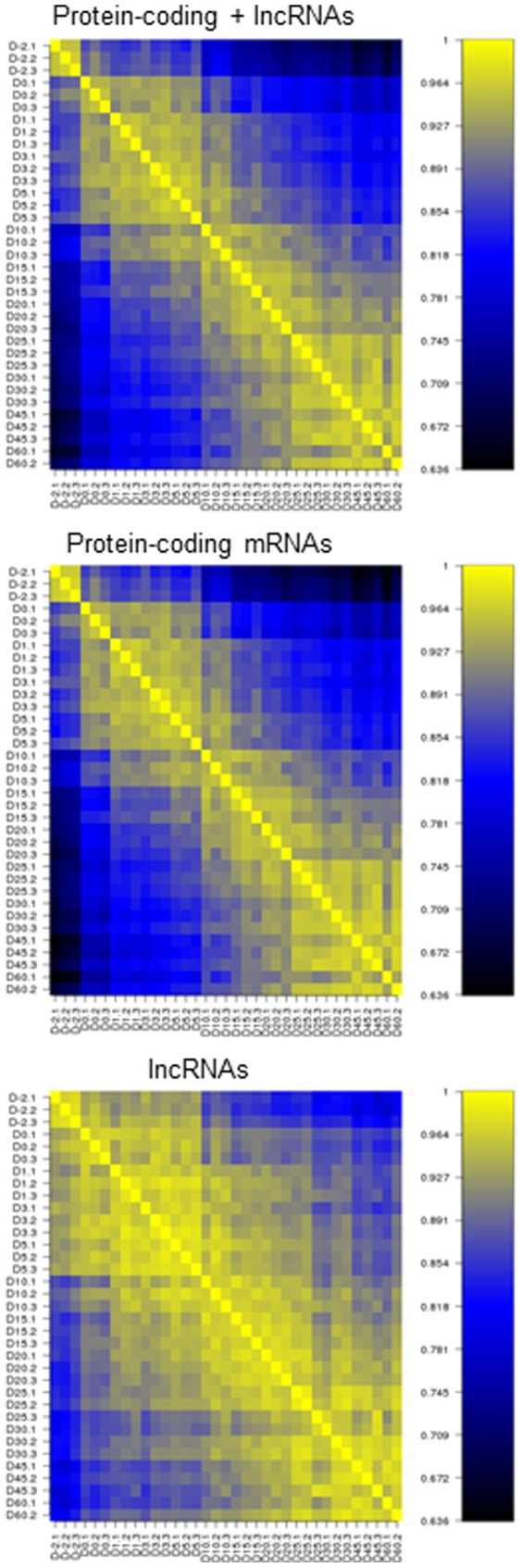
The similarity of gene expression profiles between any two different ages represented by Pearson Correlation Coefficient for protein-coding + lncRNAs, protein-coding mRNAs alone, and lncRNAs alone.

Pearson correlation coefficient between any two samples of different ages was used to assess the similarity and differences of gene expression profiles of protein-coding mRNAs + lncRNAs, protein-coding mRNAs alone, and lncRNAs alone over the 12 ages (**Fig. 2**). High correlation was found among the days, which correspond to liver development for perinatal (day −2, 0), neonatal (day 1, 3, 5), adolescent (day 15, 20, 25), and young adult (day 30, 45, 60) with significant changes between each developmental stage. Protein-coding mRNAs have a stronger similarity within each developmental stage than lncRNAs.

**Figure 3:**
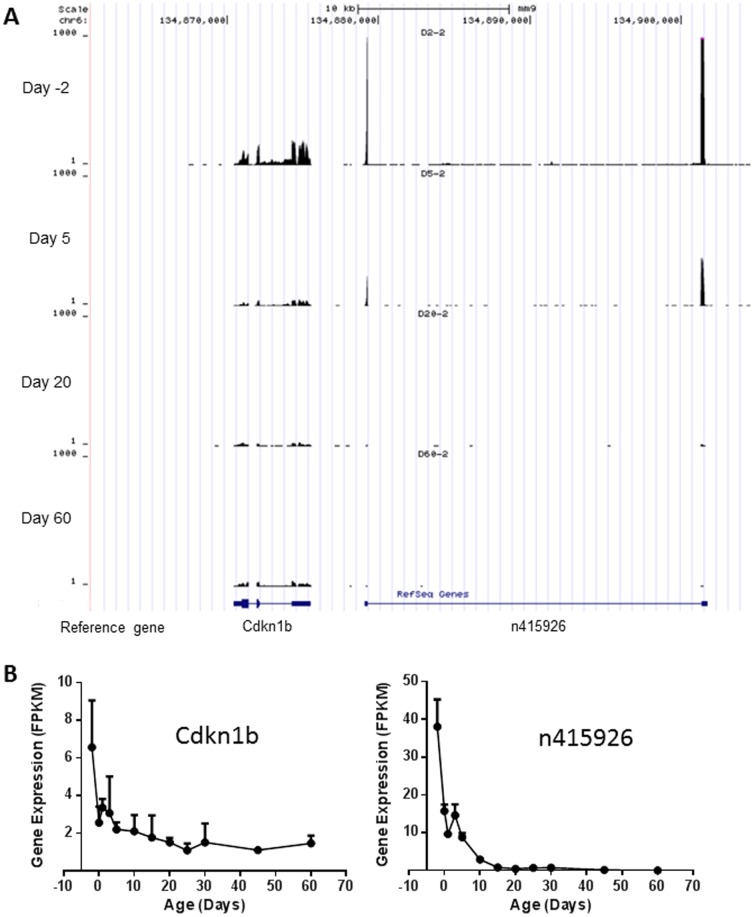
Developmental expression of lncRNA n415926 (identified by Noncode v3.0) and its neighbor protein coding gene Cdkn1b. (A) Sequence read distribution at day −2, 5, 20, and 60 viewed by the UCSC genome browser. (B) Gene expression (represented by FPKM) across the 12 selected ages.

**Figure 4:**
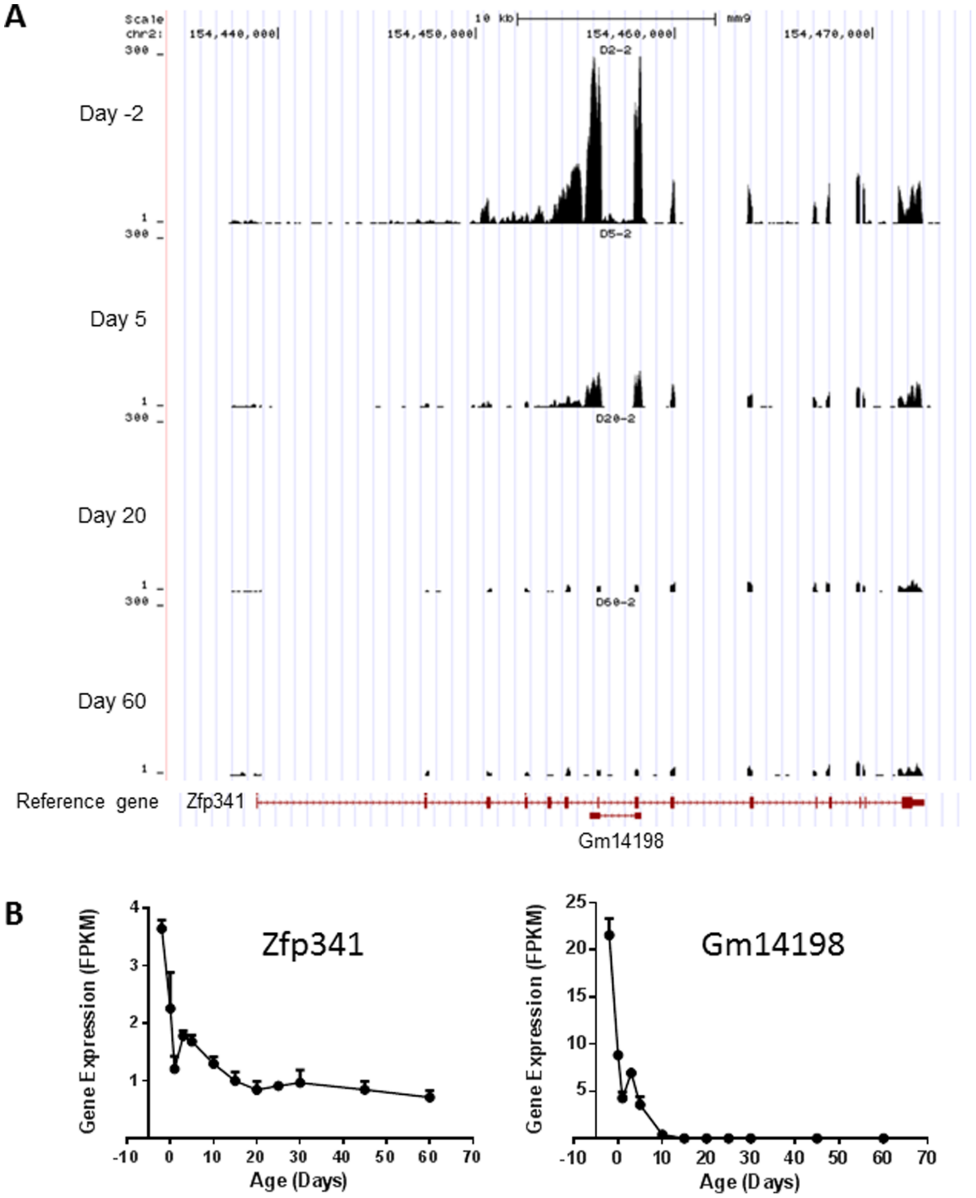
Developmental expression of antisense lncRNA Gm14198 (identified by Ensembl 66) and its neighbor protein coding gene Zfp341. (A) Sequence read distribution at day −2, 5, 20, and 60 viewed by the UCSC genome browser. (B) Gene expression (represented by FPKM) across the 12 selected ages.

**Figure 5:**
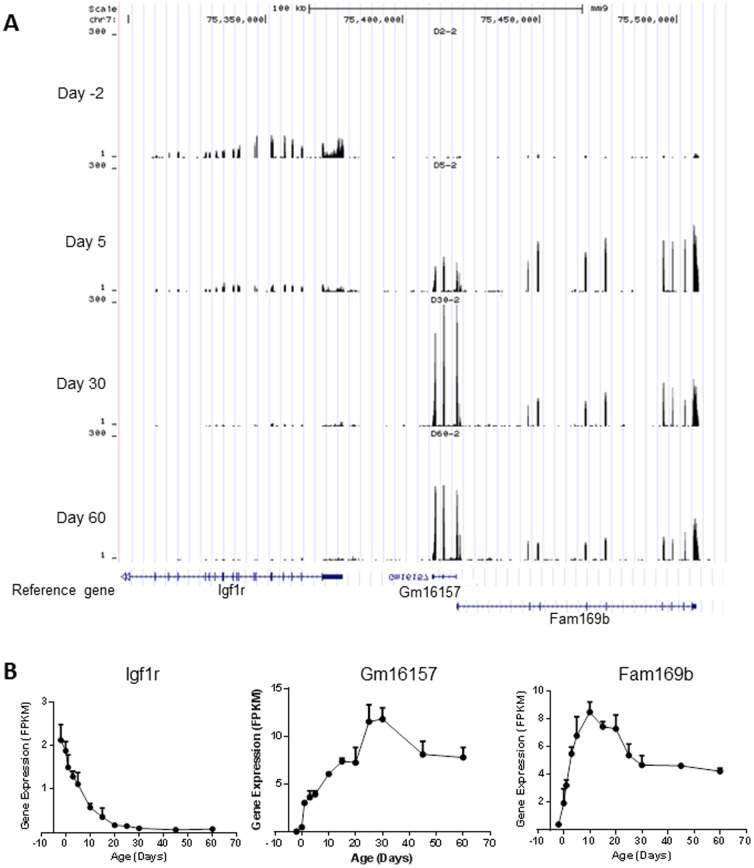
Developmental expression of lincRNA Gm16157 (identified by Ensembl 66) and its neighbor protein coding genes Fam169b and Igf1r. (A) Sequence read distribution at day −2, 5, 30, and 60 viewed by the UCSC genome browser. (B) Gene expression (represented by FPKM) across the 12 selected ages.

ANOVA test identified 10,768 (83% of the 12,990) expressed protein-coding genes and 1,450 (75% of the 1,932) expressed lncRNAs, which had significantly differential expression levels (*p*<0.05) during postnatal liver development (**Fig. 1B**). Among the 1,450 differentially expressed lncRNAs, 498 were identified by Noncode v3.0 annotation database (a full list of the lncRNAs is presented in **S2 Table**), but no biotype information is available. As an example, developmental expression of lncRNA n415926 and its neighbor gene Cdkn1b is shown in **Fig. 3**. lncRNA n415926 is located at chromosome 6 with two exons separated about 22 kb. Its neighbor coding gene Cdkn1b is located at about 3.5 kb upstream and has 3 exons. Both lncRNA n415926 and Cdkn1b decreased their expression through liver development. In addition to the 498 lncRNAs identified by Noncode v3.0, Ensembl66 annotation database identified a total of 952 differentially expressed lncRNAs with biotype identification, including processed transcripts, antisense, lincRNA, ncRNA host, noncoding, and retained intron. As an example, a full list of 106 antisense lncRNAs is presented in **S3 Table**. Developmental expression of a typical antisense Gm14198 and its complementary mRNA Zfp341 is shown in **Fig. 4**. Both Gm14198 and Zfp341 decreased their expression levels through liver maturation. As many other antisense lncRNAs founded in mouse genome [17], [32], the antisense lncRNA Gm14198 is concordant with the associated coding gene Zfp314. Another major type of lncRNA is long intergenic non-coding RNAs (lincRNAs), which are transcribed from non-coding DNA sequences between protein-coding genes. **S4 Table** lists 155 lincRNAs, which are differentially expressed during liver maturation. As an example, **Fig. 5** shows developmental expression of lincRNA Gm16157 and its downstream neighbor coding gene Fam169b and upstream neighbor coding gene Igf1r. Expression of both lincRNA Gm16157 and coding gene Fam169b is enriched at adolescent age, whereas Igf1r is enriched at prenatal and neonatal ages. Ontogenic patterns of Gm14198, Gm16157, and another lncRNA Gm12839 were validated by RT-PCR with a high correlation coefficiency (*r* value between 0.94 and 0.96 ) existing between RNA-Seq and RT-PCR (S2 Figure).

**Figure 6:**
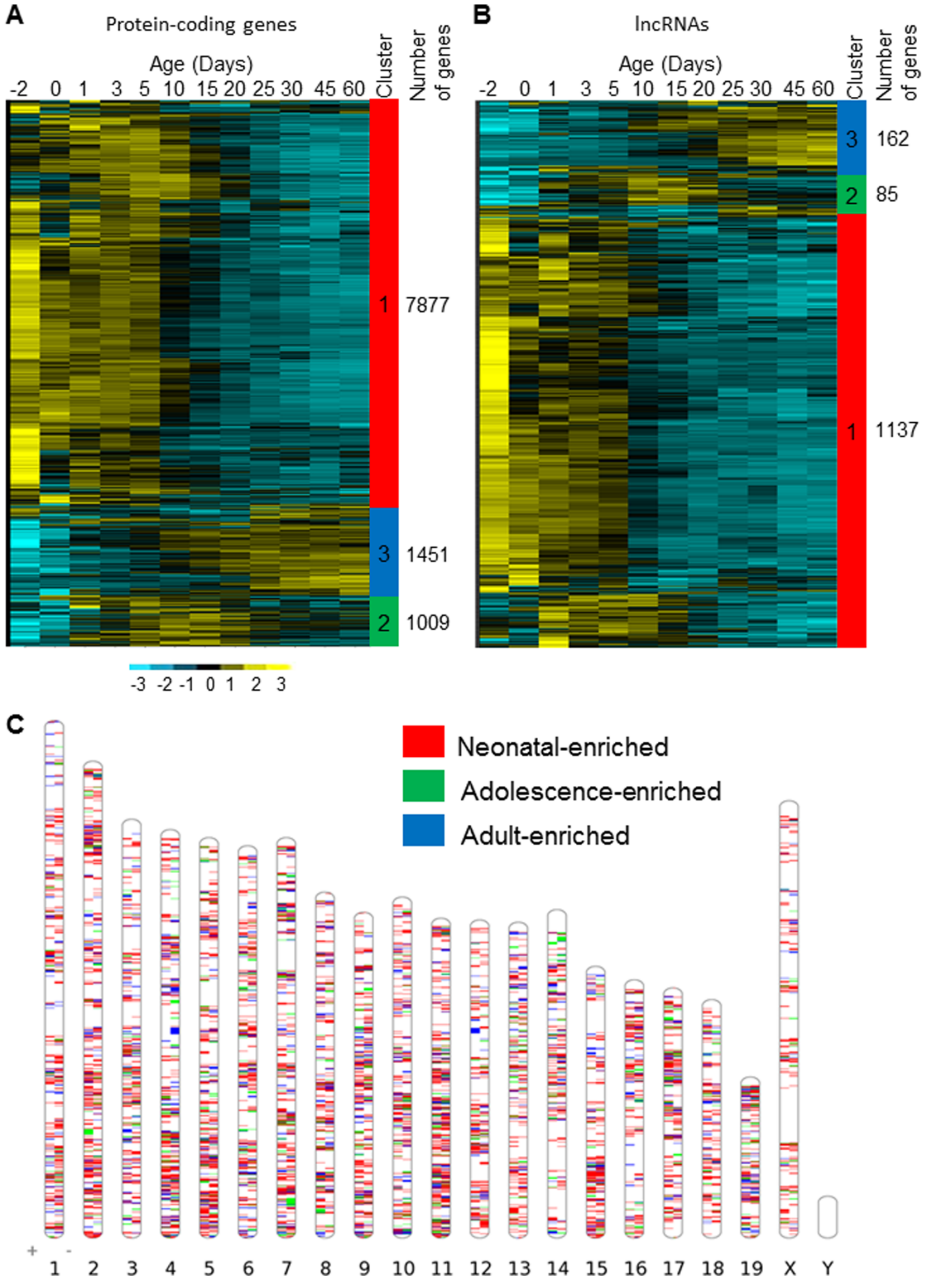
Developmental gene expression patterns during postnatal liver maturation. Heat maps are drawn for all significantly differentiated expressed protein-coding (A) and lncRNA (B) genes during development in liver. Differential expressions were detected by ANOVA with adjusted *p*-values <0.05. For each gene, log_2_(FPKM+1) over the ages were converted to z-scores, and the scale bar indicates z-score values. Genes are hierarchically clustered, and the color bar on the right shows the major trends of gene expression: high relative expression at early ages (neonatal, Cluster 1), intermediate ages (adolescent, Cluster 2) and older ages (adult, Cluster 3). (C). The ideogram of the chromosomal location of genes expressed in the three major developmental patterns.

Hierarchical clustering analysis was performed on all differentially expressed coding and non-coding genes to reveal their developmental expression patterns. Three major patterns were identified for both protein-coding (**Fig. 6A**) and lncRNA genes (**Fig. 6B**) with Cluster 1, enriched at neonatal ages; Cluster 2, enriched at adolescent ages; and Cluster 3, enriched at adult ages. The proportion of genes belong to each pattern group was similar between protein-coding and lncRNA genes, with Cluster 1 as the largest group (>70% of differentially expressed genes), followed by Cluster 3 and Cluster 2. Genes in these three groups together accounted for over 95% of all differentially expressed genes. **Fig. 6C** shows distribution of the three major patterns along each chromosome on both plus and minus strains.

#### Correlation of expression patterns between lncRNAs and their neighborhood protein-coding genes

**Figure 7:**
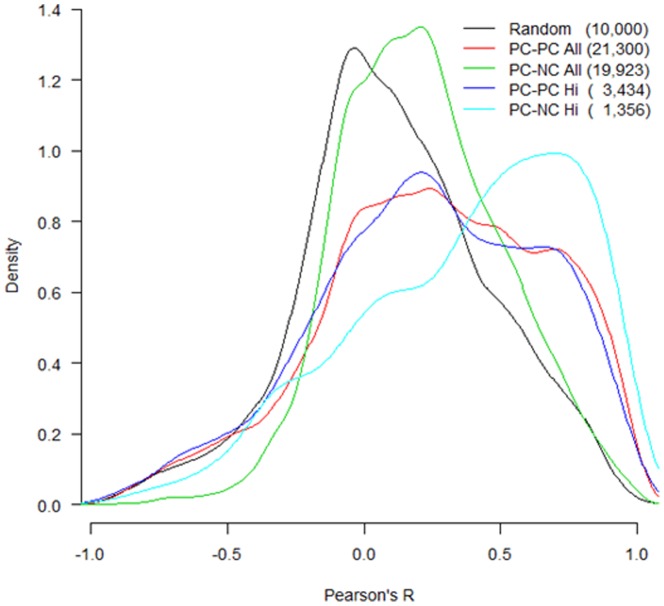
Distribution of correlation *R* value for expression patterns between pairs of neighboring genes. Random are gene pairs selected at random; PC-PC All means every nearest PC (protein-coding) neighbor for every PC gene; PC-NC All means every nearest PC neighbor for every NC (long non-coding) gene; PC-PC Hi means PC-PC neighbor pairs within 10 kb of each other, and at least one gene in the pair is expressed; PC-NC Hi means PC-NC neighbor pairs within 10 kb of each other, and NC must be expressed. The number of gene pairs in each group is shown in the parenthesis.

The emerging molecular functions of lncRNAs include regulation of gene expression in *cis* (on neighboring genes) or in *trans* (distantly located genes) [15]. One hypothesis from the in *cis* regulation in our samples is that the expression of lncRNAs and their neighboring gene loci should be correlated across the developmental ages to share the same cluster patterns. To test this hypothesis, we analyzed the correlation of ontogenic expression patterns between pairs of neighboring genes by examining the distribution of their correlation *R* values (**Fig. 7**). As a control, the distribution of *R* value for 10,000 random selected protein-coding gene (PC) pairs showed a peak around 0 (no correlation). Since the correlation of expression between a lncRNA (NC) and its neighbor PC gene may result from a true *cis* effect of the NC on its PC neighbor, or merely proximal transcriptional activity in the surrounding open chromatin, we also compared the correlation between neighboring PC-PC and PC-NC pairs. Two different stringency levels for selecting neighboring pairs were used. The lower stringency for PC-PC selected 23,232 pairs, including every nearest PC neighbor for every PC gene without filters, and the lower stringency for PC-NC pairs selected 27,594 pairs, including every nearest PC neighbor for every NC gene. The higher stringency limited neighboring gene pairs to a 10 kb distance from each other, and at least one gene must be expressed, which reduced the number of pairs to 3,434 for PC-PC and 1,356 for PC-NC. At low stringency, the distribution of correlation *R* for both PC-PC and PC-NC pairs showed a peak around 0.2. At high stringency, which is a more reasonable criterion, the distribution peak of *R* values for PC-NC was around 0.8, while that for PC-PC was still around 0.2. This result demonstrated that PC-NC neighboring genes tended to exhibit high correlation in ontogenic expression patterns.

We identified 433 pairs of PC-NC, in which lncRNAs were differentially expressed during liver maturation (adjusted *p*-value <0.05 in ANOVA analysis) and their neighboring protein-coding genes with a distance less than 10 kb were expressed in the same ontogenic patterns as the lncRNAs (**S5 Table**). GO analysis for the neighboring protein-coding genes (**Table 2**) revealed that major pathways for neonatal enriched patterns are associated with cellular component organization, cellular metabolic process, chromosome segregation and mitosis, cell cycle regulation, DNA repair, hematopoiesis, and macromolecular biosynthesis and localization. In contrast, adolescent enriched pathways are mainly related to metabolic processes, including metabolism of monocarboxylic acids, fatty acids, cellular ketones, lipids, and thioesters.

**Table 2:** Representative GO categories for protein-coding genes with neighboring lncRNAs expressed in the same developmental patterns.

Pattern	GO category	False Discovery Rate
Neonatal enriched	Cellular component organization	0
	Cellular metabolic process	0
	Mitosis	0
	Chromosome segregation	0
	Regulation of cell cycle process	0
	Macromolecule biosynthetic process	0.002
	DNA replication	0.018
	Hematopoiesis	0.043
	Macromolecule localization	0.046
Adolescent enriched	Monocarboxylic acid metabolic process	0
	Fatty acid metabolic process	0
	Cellular ketone metabolic process	0.005
	Lipid metabolic process	0.027
	Thioester metabolic process	0.029
	Lipid transport	0.038
	Regulation of cytoskeleton organization	0.040

## Discussion

In this study, we used RNA-Seq to quantify lncRNA expression levels in mouse liver samples across various ages from prenatal, through neonatal and adolescent to adult during liver maturation, in which liver functions are significantly changed from a hematopoietic organ to a metabolic organ. A poly-T selected approach was used to generate sequencing libraries for RNA-Seq, which might lead to miss quantification of some lncRNAs and small non-coding RNAs, but major lncRNAs with poly-A tails should be caught by the method. As other previous findings in zebrafish and humans [11], [13], lncRNAs were expressed at a lower level than protein coding genes (average ∼10 fold lower) in all mouse liver samples we examined, suggesting a general property of lncRNAs.

By using a same cutoff at FPKM>1 as protein coding genes, we identified 1,932 lncRNAs expressed in mouse liver, of which 1,450 (75% of the 1,932) had significantly differential expression levels (*p*<0.05) during postnatal liver maturation. The lists of several biotypes of lncRNAs with differential expression levels (*p*<0.05), such as antisense and lincRNAs, have provided a basic information for searching significantly differential expressed lncRNAs in liver during maturation.

Non-coding RNAs have been found to be pervasively expressed in the genome, and lncRNAs are an important class involved in a variety of biological functions. The molecular mechanisms for lncRNAs to regulate gene expression are largely base on their ability to form complex secondary structures and specifically interact with proteins like transcription factors and chromatin modifiers, either blocking or facilitating their activity in transcription [15]. Our result of lncRNA ontogenic expression patterns (**Fig. 6B**), which was similar as protein-coding RNAs, strongly suggested the implication of lncRNA in regulation of hepatic gene transcription during postnatal development. Among the three major ontogenic patterns identified in lncRNAs, more lncRNAs enriched at neonatal ages than at adolescent or adult ages (Fig. 6B). A similar distribution of the three ontogenic patterns is also observed in protein coding RNAs (Fig. 6A). These results may reflect functional transition during postnatal liver maturation. At neonatal ages, liver cells are active for cell proliferation and growth. Many genes involved in the cell growth processes have a higher expression level at neonatal ages. When liver becomes mature, cell proliferation processes slow down and genes related to more specific functions, such as metabolism, increase expression levels. Numbers of genes in the three clusters may reflect more general cellular functions at neonatal ages and more specific metabolic functions at adult ages. Neighboring protein-coding and lncRNA pairs exhibited higher correlations in ontogenic expression than neighboring protein-coding gene pairs under the same selection criteria, indicating that in *cis* regulation by lncRNAs may be an important mechanism of developmental gene expression in mouse liver. More than 400 pairs of non-coding RNAs and their neighbor protein coding genes with same ontogenic expression patterns have been identified (**S5 Table**). The pathway analysis of the neighborhood protein-coding genes indicates that the identified lncRNAs may involve in the functional transition of liver from neonatal hematopoietic organ to adult metabolism organ. This was the first attempt to explore the potential role of lncRNAs in liver maturation, which clustered lncRNAs into different pattern groups and provided foundations and clues for study of their molecular functions in more details.

## Supporting Information

S1 Figure **Distribution of RNA expression for protein-coding genes and lncRNAs from the age of day 5 as an example.** The FPKM value for each gene was the average FPKM of three individual animals.
(EPS)

S2 Figure **Validation of lncRNA ontogenic patterns by RT-PCR.** Relative expression levels of lncRNAs were normalized to TBP (TATA Box Binding Protein).
(EPS)

S1 Table **Expression of all protein-coding genes and lncRNAs annotated by Ensambl66 and Noncode v3.0 in liver samples at day −2, 0, 1, 3, 5, 10, 15, 20, 25, 30, 45, and 60 (n = 3).**
(XLSX)

S2 Table **Significantly differentially expressed lncRNAs annotated by Noncode v3.0 during liver maturation.**
(XLSX)

S3 Table **Significantly differentially expressed antisense lncRNAs annotated by Ensembl66 during liver maturation.**
(XLSX)

S4 Table **Significantly differentially expressed lincRNAs annotated by Ensembl66 during liver maturation.**
(XLSX)

S5 Table **Pairs of lncRNAs and their neighborhood protein coding genes with same ontogenic expression patterns during liver maturation.**
(XLSX)
